# Global change, life‐history complexity and the potential for evolutionary rescue

**DOI:** 10.1111/eva.12396

**Published:** 2016-06-30

**Authors:** Dustin J. Marshall, Scott C. Burgess, Tim Connallon

**Affiliations:** ^1^ Centre for Geometric BiologyMonash UniversityMelbourneVic.Australia; ^2^ School of Biological SciencesMonash UniversityMelbourneVic.Australia; ^3^ Department of Biological ScienceFlorida State UniversityTallahasseeFLUSA

**Keywords:** evolutionary theory, life history evolution, quantitative genetics

## Abstract

Most organisms have complex life cycles, and in marine taxa, larval life‐history stages tend to be more sensitive to environmental stress than adult (reproductive) life‐history stages. While there are several models of stage‐specific adaptation across the life history, the extent to which differential sensitivity to environmental stress (defined here as reductions in absolute fitness across the life history) affects the tempo of adaptive evolution to change remains unclear. We used a heuristic model to explore how commonly observed features associated with marine complex life histories alter a population's capacity to cope with environmental change. We found that increasing the complexity of the life history generally reduces the evolutionary potential of taxa to cope with environmental change. Our model also predicted that genetic correlations in stress tolerance between stages, levels of genetic variance in each stage, and the relative plasticity of different stages, all interact to affect the maximum rate of environmental change that will permit species persistence. Our results suggest that marine organisms with complex life cycles are particularly vulnerable to anthropogenic global change, but we lack empirical estimates of key parameters for most species.

## Introduction

Marine organisms face myriad stressors associated with anthropogenic climate change, chief among these being increases in environmental temperature and decreases in seawater pH (Pandolfi et al. 2011). Future global change is likely to reduce the viability of populations across a broad range of taxa if it reduces survival, growth and reproduction (Parmesan 2006). However, if the traits that influence survival, growth and reproduction change rapidly in response to the selection exerted by global change, then extinction risk might be reduced. The process whereby rapid (i.e. on the timescale relevant to demographic rates) adaptive evolution of life‐history traits prevents population extinction in stressful environments is known as evolutionary rescue (Gomulkiewicz and Holt 1995; Chevin and Lande 2010; Chevin et al. 2013). Changes in life‐history traits over time may also be facilitated by adaptive phenotypic plasticity, where individuals change their phenotype within or between generations in the direction favoured by selection in the new environment. Complicating matters further is the possibility for evolutionary change in plasticity itself (Chevin and Lande 2010). Critically, we still have little understanding of how phenotypic plasticity and genetic evolution buffer species from extinction in the face of environmental change (Bell 2013). A key uncertainty is whether evolution will proceed rapidly enough to keep up with the unprecedented pace of anthropogenic change (Munday et al. 2013).

As a first step towards understanding the limits to evolutionary rescue, several authors have generated a series of models that theoretically explore how phenotypic plasticity, evolution and rapid environmental change affect population persistence (Baskett et al. 2010; Chevin and Lande 2010; Chevin et al. 2010; Bell 2013). While general models of evolutionary rescue have been invaluable to understanding the potential for species to cope with climate change more generally, they provide less insight into marine organisms specifically. Marine organisms have a number of characteristics that alter the way evolution might proceed in response to climate change, such that general models of phenotypic plasticity and evolution are unlikely to capture all their dynamics.

Most marine organisms have complex life cycles, with early larval stages in pelagic habitats that are morphologically and functionally distinct from later adult stages in benthic habitats. Complex life cycles – particularly those characterized by drastic differences between pre‐ and postreproductive stages – may complicate responses to selection relative to the responses in species with more simple life histories (Moran 1994; Marshall and Morgan 2011). For example, if traits in different life‐history stages are genetically correlated, the vectors of response to selection in each will differ relative to if those traits were independent (Marshall and Morgan 2011). In general, theory predicts that evolutionary responses will be slower and more constrained in species with complex life histories than in species with simple life histories (Schluter et al. 1991). However, this prediction depends strongly on the direction of selection relative to genetic (co)variance: in some instances, adaptation can be facilitated by complementary selection pressures acting across multiple life‐history stages (Schluter et al. 1991). In other instances, genetic covariances can constrain adaptation (Schluter et al. 1991; Agrawal and Stinchcombe 2009).

Marine life‐history stages are not evolutionary independent units; strong genetic and phenotypic links constrain evolution in each (Marshall and Morgan 2011). For example, in the ascidian *Ciona intestinalis*, there is little evolutionary potential for survival in both the larval and adult stage to be maximized simultaneously – evolution of increased survival in one stage reduces survival in others (Aguirre et al. 2014). Some links between life‐history stages are rather subtle: in a marine worm, experimentally induced evolution in an early life‐history stage (egg size) yielded correlated responses in sex allocation in the adult stage (Miles and Wayne 2009). Such correlations between life‐history stages mean that evolutionary responses in one stage are likely to have ramifications for another, altering the pace and direction of evolution in response to global change.

The complex life histories in marine organisms further complicate predictions about evolutionary potential because of the vast distances early life‐history stages can travel relative to other terrestrial organisms and because of the differential sensitivity of adults and larvae to environmental stress in marine systems. Marine embryos and larvae can spend minutes, days or even months in the plankton (Shanks et al. 2003). Dispersal distances of early life‐history stages are generally greater and more variable in marine species relative to terrestrial species (Kinlan and Gaines 2003). Thus, adults and larvae can occupy very different habitats. For example, adults may spend their entire life in coastal habitats near the sea floor, while their larvae spend most of their lives in the water column. Many species (around 50% of invertebrates for which there are data) must feed in the plankton as larvae to complete development (Marshall et al. 2012). Some species living in the deep sea (the largest habitat on the planet) must move to productive surface waters as larvae (Arellano et al. 2014). Conversely, intertidal adults must endure extreme temperatures while exposed at low tide while their larvae experience relatively more buffered conditions. The adults and larvae of marine organisms are therefore likely to experience very different thermal environments. For example, some molluscs experience stable temperatures of 8°C as adults, while their larvae experience and survive in variable temperatures with a mean of around 25°C (Arellano et al. 2014). For such species, their experience of future changes in temperature will also be very different compared to species in which the larval and adult stages experience similar thermal environments. While temperatures are rising at all ocean depths, the surface waters are warming more quickly (Levitus et al. 2012), such that pelagic larvae are likely to experience more rapid environmental change than their benthic adult counterparts (although it is worth noting that intertidal species may experience the converse). Thus, selection to evolve greater tolerances to higher temperature will be very different for adult and larval stages of the same organism. The extent to which stage‐specific selection associated with global change affects predictions about evolutionary rescue remains unclear.

Early life‐history stages of marine organisms are especially vulnerable to stress. Marine eggs and larvae are typically tiny (most are much less than 500 μm in diameter, and many are less than 100 μm; Marshall et al. 2012). Around half of all marine invertebrates and many species of fish have external fertilization, whereby eggs and sperm are shed directly into the water column whereupon fertilization occurs when eggs and sperm fuse, after which embryonic development occurs entirely externally (Kasimatis and Riginos 2015; Monro and Marshall 2015). The small size of eggs, sperm and larvae means they have large surface to volume ratios. Larvae are also often anatomically simpler than adults, with fewer organs for maintaining physiological stasis and thinner integuments for buffering their tissues from external stressors. The relative size and simplicity of eggs and larvae make them much more sensitive to environmental stress generally and global change stressors specifically (Byrne 2011, 2012; Przeslawski et al. 2015). Temperature changes that are relatively benign to adults can be catastrophic for larvae and adult marine organisms seem better able to cope with changes in water chemistry (Przeslawski et al. 2015). Thus, while some species have larvae that can tolerate extremely wide temperature ranges relative to adults (Arellano et al. 2014), meta‐analyses suggest that on average, early life‐history stages are more sensitive to change than later stages. Given the differences in the sensitivity of adult and early life‐history stages to global change, initial changes in thermal regimes may not affect the fitness of adults, but these same changes will induce massive shifts in selection in the larval stage. How these differences in the relative sensitivity of adults and larvae alter the evolutionary capacity of species as a whole remains unclear, although some reviews suggest that the increased sensitivity of early life‐history stages creates particular vulnerabilities and demographic bottlenecks for marine organisms (Byrne 2012; Przeslawski et al. 2015).

The ubiquity of complex life cycles in marine taxa means that an explicit consideration of the evolutionary potential of complex life histories to cope with global change is necessary. Complex patterns of environmental variation, stage‐specific selection and genetic covariances have recently been accommodated within models stage‐specific adaptation (Barfield et al. 2011; Cotto and Ronce 2014), as has the evolution of phenotypic plasticity across life‐history stages (Fischer et al. 2014). These studies provide a rigorous framework for modelling the evolution of traits within stage‐structured populations. Nevertheless, we still lack a model that simultaneously combines arbitrary stage‐specific selection (including differential sensitivity between larvae and adults to environmental change), stage‐specific plasticity and genetic correlations within an explicit context of evolutionary rescue that is pertinent to marine organisms. Our aim here is to develop a simple, heuristic model that considers how commonly observed features associated with marine complex life histories (genetic correlations between ecologically and morphologically distinct larval and adult stages, differential selection pressures among stages, differing degrees of plasticity among stages and differing levels of genetic variation among stages) alter the capacity to cope with environmental change.

We wish to acknowledge that our model explores little in the way of new theory; instead, most of our findings can be reconstructed combining elements from several other theory papers (with a few necessary tweaks), so we should clarify our goal and audience here. Our intended audience here are empiricists with a specific interest in estimating the potential for marine organisms to adapt and cope with global change but may be less familiar with the general theoretical literature on the interplay between adaptation, life history and demography. In our experience, it is often difficult for empiricists to take these general models and apply them specifically to their system. Given our audience, our goal is not to advance theory but instead provide an integrated and explicit treatment of the issues that are most relevant to empiricists working on marine organisms with complex life histories, particularly species with highly distinctive larval and adult stages. Our approach is to apply general models to a specific problem so as to identify and prioritize key gaps in our understanding for marine biologists interested in global change. Consequently, we anticipate theoreticians will find some of our explorations redundant because they may intuit some regions of the parameter space based on the equations provided, but we provide these explorations in the hope that they will identify key areas that are worthy of empirical exploration. Wherever possible, we refer to the general theoretical studies from which we sourced our model components to assist that those reader wishing to explore these topics more deeply. As first step, those with a deep theoretical interest in this topic should explore Lande (1979), Gomulkiewicz and Houle (2009), Barfield et al. (2011), Chevin (2013), Cotto and Ronce (2014) and Kopp and Matuszewski (2014) as the foundational papers that explore the general concepts of adaptation to environmental change.

## Model

We consider a simple model of demography and evolutionary change in a species with two dominant life‐history stages: a larval stage and an adult stage. In an effort to maintain simplicity, and because our key point of contrast is between reproductive and prereproductive stages, we do not consider overlapping generations, which compartmentalize selection between different reproductive age classes. For theoretical considerations of life‐history complexity within (st)age‐structured models, we recommend Lande (1982), Ellner and Rees (2006), Coulson and Tuljapurkar (2008), Barfield et al. (2011), and Cotto and Ronce (2014).

Our model draws heavily from the highly influential framework for evolutionary persistence that was developed in Chevin et al. (2010), which we have expanded by permitting the two stages to differ in their degrees of plasticity, their patterns of quantitative genetic variance and covariance and the relationship in each stage between trait expression and fitness (i.e. their fitness functions). We consider the persistence of a population that is forced to either adapt to a directional change in its environment over time, or be driven to extinction. Following Chevin et al. (2010), we assume that persistence hinges upon a key ecological trait with constant genetic and phenotypic variability. Plasticity is determined by stage‐specific linear reaction norms, which are assumed to be fixed and invariant over time. Importantly, other theoretical treatments also allow plasticity to evolve (e.g. Via and Lande 1985; Lande 2009; Chevin and Lande 2010; Reed et al. 2010; Childs et al. 2016). We chose not to here for two reasons. First, the empirical resolution of how plasticity affects evolution and persistence remains poorly resolved, plasticity can either facilitate or retard evolution and persistence depending on the underlying genetic mechanisms (Price et al. 2003). Second, our models focus on climate change, where we expect gradual shifts in average environmental conditions. In this case, most of the plastic response to changes in the environment will be *via* reaction norms that were selected in ancestral environmental contexts (e.g. reaction norms that are adaptive within the normal range of variability of ancestral environments). While the evolution of reaction norms may profoundly impact persistence in the context of abrupt environmental change (see, e.g. Chevin and Lande 2010), allowing plasticity to evolve would introduce significant additional complexity to our model. For simplicity, we keep plasticity fixed. We simply note that, as in Chevin and Lande (2010), populations should be able to tolerate more rapid environmental change when plasticity is permitted to evolve. In this instance, our results can be interpreted as a conservative baseline, a worst‐case scenario.

We consider a population evolving under a life cycle that shifts between two distinct environments of selection: a larval environment (referred to using L subscripts), and an adult environment (referred to using A subscripts). We model the evolution of breeding values for a trait that is genetically correlated between larval and adult stages. Let *x* represents the breeding value for the larval trait, and *y* the breeding value for the adult trait. The mean and variance of breeding values for the larval trait are *E*(*x*) and *G*_L_ = var(*x*), and for the adult trait is *E*(*y*) and *G*_A_ = var(*y*). The covariance in breeding values between the larval and adult trait is cov(*x*, *y*). The distribution of breeding values is assumed to follow a bivariate normal distribution, with the between stage genetic correlation represented by *ρ* = cov(*x*, *y*)[var(*x*)var(*y*)]^−0.5^. Following standard idealized assumptions of the infinitesimal model of quantitative genetics (Lande 1979, 1980), patterns of genetic and phenotypic variance are assumed to remain constant over time, including between stages – a useful approximation that is most applicable under weak stabilizing selection to an optimum (Bulmer 1980; Cotto and Ronce 2014).

### Variation and selection in larvae

The environment changes at a constant rate *η*, with time in generations (assumed to be discrete and nonoverlapping) (e.g. the rate of change in temperature in °C between generations). In a random individual at generation *t*, the larval phenotype *P*_L_, prior to selection in the larval stage, is:

$$P_{L}=x+b_{L}\eta t+e_{L},$$

where *b*_L_ is the slope of the larval reaction norm (the degree of plasticity in the larval phenotype), and $e_{L}∼N\left(0,\sigma_{L}^{2}\right)$ represents the residual environmental variability in the trait. Consequently, the mean and variance (respectively) of larval phenotypes are:

$$E\left(P_{L}\right)=E\left(x\right)+b_{L}\eta t,$$

and

$$\text{var}\left(P_{L}\right)=\text{var}\left(x\right)+\sigma_{L}^{2}.$$

Changes in the mean breeding values due to selection in the larval stage depend on the variance in breeding values in larvae, *G*_L_, the covariance in breeding value between the larval and adult stage, cov(*x*, *y*), and the directional selection gradient among larvae, *β*_L_:

(1) $$\begin{array}{cc} & \Delta {\overset{¯}{x}}_{L}=G_{L}\beta_{L}\\ & \Delta {\overset{¯}{y}}_{L}=\text{cov}\left(x,y\right)\beta_{L} \end{array}.$$

The probability of survival through the larval stage depends on the expressed phenotype *P*_L_ and is described by the larval fitness function *W*_L_(*P*_L_), which is determined by the maximum survival probability to adult (*C*_L_, i.e. for an individual that expressed a perfectly adapted phenotype with respect to the environment), the concavity of the fitness landscape for larvae (*ω*_L_) and the optimal phenotype that maximizes larval survival (*θ*_L_):

$$W_{L}\left(P_{L}\right)=C_{L}\exp\left(-\frac{{\left(\theta_{L}-P_{L}\right)}^{2}}{2\omega_{L}^{2}}\right).$$

Averaging over the distribution of larval phenotypes provides the mean survival of larvae, and the probability that each transition to the adult stage:

$${\frac{}{W}}_{L}=\int_{-\infty }^{\infty }W_{L}\left(P_{L}\right)f\left(P_{L}\right)dP_{L}=\frac{C_{L}\sqrt{\omega_{L}^{2}}}{\sqrt{\omega_{L}^{2}+\text{var}\left(P_{L}\right)}}\exp\left(-\frac{{\left(\theta_{L}-{\overset{¯}{P}}_{L}\right)}^{2}}{2\omega_{L}^{2}+2\text{var}\left(P_{L}\right)}\right),$$

where the overbars refer to means. The selection gradient, *β*_L_, is then calculated from ${\bar{W}}_{L}$ as:

$$\beta_{L}=\frac{\partial \ln({\bar{W}}_{L})}{\partial \overset{¯}{x}}=\frac{\left(\theta_{L}-{\overset{¯}{P}}_{L}\right)}{\omega_{L}^{2}+\text{var}\left(P_{L}\right)}.$$

Incorporating *β*_L_ into eqn (1), the change in the mean breeding values due to selection in the larval stage is then:

(2a) $$\Delta {\overset{¯}{x}}_{L}=\frac{G_{L}\left(\theta_{J}-{\overset{¯}{P}}_{L}\right)}{\omega_{L}^{2}+\text{var}\left(P_{L}\right)}$$

and

(2b) $$\Delta {\overset{¯}{y}}_{L}=\frac{\text{cov}\left(x,y\right)\left(\theta_{L}-{\overset{¯}{P}}_{L}\right)}{\omega_{L}^{2}+\text{var}\left(P_{L}\right)}$$

### Variation and selection in adults

The time spent in the larval stage represents a fraction *τ* of each generation. Thus, at generation *t*, the adult stage begins at time *t *+ *τ*. The adult phenotype, *P*_A_, for an individual at generation *t* is:

$$P_{A}=y^{\prime }+b_{A}\eta \left(t+\tau \right)+e_{A},$$

where *b*_A_ is the slope of the reaction norm (the degree of plasticity in the adult phase), *y′* represents the breeding value for the adult trait (the prime denotes that its distribution has been altered following selection in the larval stage), and $e_{A}∼N\left(0,\sigma_{A}^{2}\right)$ is residual environmental variability in adults. We assume that the trait in the adults is assumed to respond plastically to the same environmental variable as the larvae but not to be influenced by the plastic response in the larvae. The mean adult phenotype at generation *t* is:

$$E\left(P_{A}\right)=E\left(y^{\prime }\right)+b_{A}\eta \left(t+\tau \right),$$

and the variance in adult phenotype is:

$$\text{var}\left(P_{A}\right)=\text{var}\left(y^{\prime }\right)+\sigma_{A}^{2}=G_{A}+\sigma_{A}^{2}.$$

Because we assume the variance of breeding values remains approximately constant over time (due to weak stabilizing selection causing little change within a generation), var(*y′*) = var(*y*) = *G*_A_. The mean breeding values in the adult population, prior to selection among adults, are:

$${\overset{¯}{x}}^{\prime }=\overset{¯}{x}+\Delta {\overset{¯}{x}}_{L}=\overset{¯}{x}+\frac{G_{L}\left(\theta_{L}-{\overset{¯}{P}}_{L}\right)}{\omega_{L}^{2}+\text{var}\left(P_{L}\right)},$$

and

$${\overset{¯}{y}}^{\prime }=\overset{¯}{y}+\Delta {\overset{¯}{y}}_{L}=\overset{¯}{y}+\frac{\text{cov}\left(x,y\right)\left(\theta_{L}-{\overset{¯}{P}}_{L}\right)}{\omega_{L}^{2}+\text{var}\left(P_{L}\right)}.$$

Note that the terms $\Delta {\overset{¯}{x}}_{L}$ and $\Delta {\overset{¯}{y}}_{L}$ describe differences between mean breeding values of adults (before selection adults) differ from the mean breeding values of larvae (before selection in larvae).

The change in the breeding values due to selection in the adult stage is:

(3) $$\begin{array}{cc} & \Delta {\overset{¯}{x}}_{A}=\text{cov}\left(x,y\right)\beta_{A}\\ & \Delta {\overset{¯}{y}}_{A}=G_{A}\beta_{A} \end{array},$$

where *β*_A_ is the directional selection gradient among adults. Trait expression can influence any combination of survival and fecundity within the adult stage. Selection on adults is based on the net productivity of different phenotypes, with respect to the next generation. Individual fitness (survival + fecundity) at the adult stage (*W*_A_) depends on the adult phenotype (*P*_A_), the maximum absolute fitness in the adult stage (*C*_A_), the concavity of the adult fitness function (*ω*_A_) and the optimal adult phenotype (*θ*_A_):

$$W_{A}\left(P_{A}\right)=C_{A}\exp\left(-\frac{{\left(\theta_{A}-P_{A}\right)}^{2}}{2\omega_{A}^{2}}\right).$$

Averaging over the distribution of adult phenotypes provides the mean contribution of adults to production of offspring in the next generation:

$${\bar{W}}_{A}=\int_{-\infty }^{\infty }W_{A}\left(P_{A}\right)f\left(P_{A}\right)dP_{A}=\frac{C_{A}\sqrt{\omega_{A}^{2}}}{\sqrt{\omega_{A}^{2}+\text{var}\left(P_{A}\right)}}\exp\left(-\frac{{\left(\theta_{A}-{\overset{¯}{P}}_{A}\right)}^{2}}{2\omega_{A}^{2}+2\text{var}\left(P_{A}\right)}\right).$$

Consequently, the selection gradient for the adult stage is:

$$\beta_{A}=\frac{\partial \ln({\bar{W}}_{A})}{\partial {\overset{¯}{y}}^{\prime }}=\frac{\left(\theta_{A}-{\overset{¯}{P}}_{A}\right)}{\omega_{A}^{2}+\text{var}\left(P_{A}\right)}.$$

Incorporating *β*_A_ into eqn (3), the change in the breeding values due to selection in the adult stage is:

$$\Delta {\overset{¯}{x}}_{A}=\frac{\text{cov}\left(x,y\right)\left(\theta_{A}-{\overset{¯}{P}}_{A}\right)}{\omega_{A}^{2}+\text{var}\left(P_{A}\right)},$$

and

$$\Delta {\overset{¯}{y}}_{A}=\frac{G_{A}\left(\theta_{A}-{\overset{¯}{P}}_{A}\right)}{\omega_{A}^{2}+\text{var}\left(P_{A}\right)}.$$

### Stage‐specific patterns of evolutionary change

Across a full generation (i.e. combining selection in larval and adult stages), the change in the breeding values will be:

$$\Delta \overset{¯}{x}=\frac{G_{L}\left(\theta_{L}-{\overset{¯}{P}}_{L}\right)}{\omega_{L}^{2}+\text{var}\left(P_{L}\right)}+\frac{\text{cov}\left(x,y\right)\left(\theta_{A}-{\overset{¯}{P}}_{A}\right)}{\omega_{A}^{2}+\text{var}\left(P_{A}\right)},$$

and

$$\Delta \overset{¯}{y}=\frac{\text{cov}\left(x,y\right)\left(\theta_{L}-{\overset{¯}{P}}_{L}\right)}{\omega_{L}^{2}+\text{var}\left(P_{L}\right)}+\frac{G_{A}\left(\theta_{A}-{\overset{¯}{P}}_{A}\right)}{\omega_{A}^{2}+\text{var}\left(P_{A}\right)}.$$

The rates of change of the larval and adult phenotypes are:

$$\Delta {\overset{¯}{P}}_{L}=\Delta \overset{¯}{x}+b_{L}\eta =\frac{G_{L}\left(\theta_{L}-{\overset{¯}{P}}_{L}\right)}{\omega_{L}^{2}+\text{var}\left(P_{L}\right)}+\frac{\text{cov}\left(x,y\right)\left(\theta_{A}-{\overset{¯}{P}}_{A}\right)}{\omega_{A}^{2}+\text{var}\left(P_{A}\right)}+b_{L}\eta ,$$

and

$$\Delta {\overset{¯}{P}}_{A}=\Delta \overset{¯}{y}+b_{A}\eta =\frac{\text{cov}\left(x,y\right)\left(\theta_{L}-{\overset{¯}{P}}_{L}\right)}{\omega_{L}^{2}+\text{var}\left(P_{L}\right)}+\frac{G_{A}\left(\theta_{A}-{\overset{¯}{P}}_{A}\right)}{\omega_{A}^{2}+\text{var}\left(P_{A}\right)}+b_{A}\eta .$$

Following Chevin et al. (2010; also see Cotto and Ronce 2014), phenotypic optima are assumed to change linearly over time, whereas other parameters of larval and adult fitness functions (*C*_L_, *C*_A_, *ω*_L_, *ω*_A_) are assumed to be constant. The rates of change of larval and adult optima are (respectively) ∆*θ*_L_ = *B*_L_ *η* and ∆*θ*_A_ = *B*_A_ *η*, where parameters *B*_L_ and *B*_A_ represent the sensitivity of each optimum to changes in the environment (i.e. the rate of change in an optimum per change in the environment). Over many generations, the rates of change of mean phenotype and optimum converge, leading to a steady‐state displacement between mean stage‐specific trait values and the value of their respective optima (the ‘lag’). The equilibrium lags within larval and adult stages are:

$${\left(\theta_{L}-{\overset{¯}{P}}_{L}\right)}_{\mathrm{eq}}=\frac{\eta }{\gamma_{L}\left(1-\rho^{2}\right)}\left[\frac{\left(B_{L}-b_{L}\right)}{G_{L}}-\frac{\rho (B_{A}-b_{A})}{\sqrt{G_{L}G_{A}}}\right],$$

and

$${\left(\theta_{A}-{\overset{¯}{P}}_{A}\right)}_{\mathrm{eq}}=\frac{\eta }{\gamma_{A}\left(1-\rho^{2}\right)}\left[\frac{\left(B_{A}-b_{A}\right)}{G_{A}}-\frac{\rho (B_{L}-b_{L})}{\sqrt{G_{L}G_{A}}}\right],$$

where $\gamma_{L}=1/\left(\omega_{L}^{2}+\text{var}\left(P_{L}\right)\right)$ and $\gamma_{A}=1/\left(\omega_{A}^{2}+\text{var}\left(P_{A}\right)\right)$ represent the stage‐specific strengths of stabilizing selection near each optimum (higher *γ* means stronger stabilizing selection). A larger lag increases the risk of extinction (see below). The *net* sensitivity to environmental change in the larval and adult stage is represented by (*B*_L_ − *b*_L_) and (*B*_A_ − *b*_A_), respectively, and is the outcome of the environmental sensitivity of the optimal phenotype (*B*_L_ and *B*_A_) and the slope of the reaction norms (*b*_L_ and *b*_L_). If 0 < *b *< *B*, then higher plasticity (larger *b*) will produce a phenotype closer to the environment‐specific optima and reduce the strength of directional selection (Chevin et al. 2010). Although reduced directional selection will lead to reduced genetic response (i.e. evolutionary change), increased plasticity will help in reducing the lag overall. Placing this lag between environmental change and the population's mean phenotype into a demographic context (below) allows us to assess the degree to which phenotypic plasticity and genetic evolution buffer species from extinction in the face of environmental change.

Optima for both stages move in the same direction in our model – we explore this space only because it is most relevant to exploring the impacts of climate change – temperatures are increasing and pHs are decreasing such that both adults and larvae should experience the same direction of change. Those interested in the more general case of differences in the direction of change in optima among stage could modify our model but for now, we note that our conclusions would likely be very different were we to include such a scenario. We have assumed here that larvae are more sensitive to change than adults, but it is worth noting that the model can be equally applied to species where larvae may be more tolerant to change than adults.

### Demographic model

Demography depends on the product of larval survival and adult reproductive output, with the latter a function of adult survival and fecundity. We model population growth using the recursion:

$$N_{t+1}=N_{t}{\bar{W}}_{L}{\bar{W}}_{A}.$$

We ignore the possibility of density dependence because we are only interested in whether a population at low density will increase in abundance or continue to decline to extinction. Importantly, our conclusions about whether long‐term net growth is positive will apply whether or not there is density regulation in a population that is closer to carrying capacity. It has been shown elsewhere that inclusion of negative density dependence can dampen the potential for evolutionary rescue and population persistence (see Chevin and Lande 2010). The criterion for positive growth – and thus, persistence – is

$${\bar{W}}_{L}{\bar{W}}_{A}>1⟺\ln\left({\overset{¯}{W}}_{L}{\overset{¯}{W}}_{A}\right)>0,$$

where

$$\ln\left({\bar{W}}_{L}{\bar{W}}_{A}\right)=r_{\max}-\frac{\gamma_{L}{\left(\theta_{L}-{\overset{¯}{P}}_{L}\right)}^{2}}{2}-\frac{\gamma_{A}{\left(\theta_{A}-{\overset{¯}{P}}_{A}\right)}^{2}}{2},$$

where *r*_max_ represents the growth rate of a hypothetical population (from low abundance) that has evolved to both stage‐specific optima:

$$r_{\max}=\ln\left[\frac{C_{L}C_{A}\sqrt{\omega_{L}^{2}\omega_{A}^{2}}}{\sqrt{\left(\omega_{L}^{2}+\text{var}\left(P_{L}\right)\right)\left(\omega_{A}^{2}+\text{var}\left(P_{A}\right)\right)}}\right]=\ln\left(C_{L}C_{A}\sqrt{\gamma_{L}\gamma_{A}\omega_{L}^{2}\omega_{A}^{2}}\right).$$

At steady state, the population growth rate reaches an equilibrium:

$$\begin{array}{cc} & r_{\mathrm{eq}}=r_{\max}-\frac{1}{2\gamma_{L}}{\left(\frac{\eta }{\left(1-\rho^{2}\right)}\left[\frac{\left(B_{L}-b_{L}\right)}{G_{L}}-\frac{\rho (B_{A}-b_{A})}{\sqrt{G_{L}G_{A}}}\right]\right)}^{2}\\ & \;\;-\frac{1}{2\gamma_{A}}{\left(\frac{\eta }{\left(1-\rho^{2}\right)}\left[\frac{\left(B_{A}-b_{A}\right)}{G_{A}}-\frac{\rho (B_{L}-b_{L})}{\sqrt{G_{L}G_{A}}}\right]\right)}^{2}. \end{array}$$

Parameter conditions leading to zero growth at equilibrium (*r*_eq_ = 0) define the threshold rate of environmental change, above which the population will go extinct. The maximum rate of environmental change under which the population can still persist (i.e. maintain positive growth) is *η*_crit_. Lower *η*_crit_ means that populations are more prone to extinction than populations with higher *η*_crit_ under more rapid environmental change. *η*_crit_ depends on the between stage genetic correlation (*ρ*), the growth rate of the population from low abundance (*r*_max_), the strength of stabilizing selection (*γ*_*j*_), genetic variance in breeding values (*G*_*j*_) and *net* sensitivity of selection to environmental change (*B*_*j*_ − *b*_*j*_):

(4) $$\eta_{\mathrm{crit}}=\left(1-\rho^{2}\right)\sqrt{\frac{2r_{\max}}{\Psi }},$$

where

$$\begin{array}{cc} & \Psi =\frac{1}{\gamma_{L}}{\left(\frac{\left(B_{L}-b_{L}\right)}{G_{L}}-\frac{\rho (B_{A}-b_{A})}{\sqrt{G_{L}G_{A}}}\right)}^{2}\\ & \;\;\;\;\;+\frac{1}{\gamma_{A}}{\left(\frac{\left(B_{A}-b_{A}\right)}{G_{A}}-\frac{\rho (B_{L}-b_{L})}{\sqrt{G_{L}G_{A}}}\right)}^{2}. \end{array}$$

In the absence of any differences between stages (*B*_L_ = *B*_A_, *b*_L_ = *b*_A_, *ω*_L_ = *ω*_A_, var(*x*) = var(*y*), var(*P*_L_) = var(*P*_A_), *ρ* = 1), the critical rate of environmental change reduces to:

(5) $$\eta_{\mathrm{crit}}=\frac{2G\sqrt{r_{\max}\gamma }}{\left|B-b\right|}.$$

Equation (5) is essentially identical to that of Chevin et al. (2010, see eqn (1)), except that it is multiplied by a factor of √2. The difference reflects the fact that two bouts of selection (rather than one) occur during each generation of our model.

## Results

### Impact of complex life histories on potential for evolutionary rescue

Complex life histories partially decouple the genetic basis of shared traits (through an imperfect genetic correlation between larval and adult breeding values; *ρ* < 1) and introduce divergence between larval and adult stages with respect to their relative levels of additive genetic variation (*G*_*j*_, for the *j*th stage), the strength of stabilizing selection (*γ*_*j*_) and the net sensitivity of each stage to environmental change (*B*_*j*_ − *b*_*j*_). How then is life‐history complexity expected to alter extinction risk of populations facing environmental change? To gauge the effect life‐cycle complexity on population persistence, it is useful to contrast the relative magnitude of *η*_crit_ between simple and complex life‐cycle scenarios. We therefore calculated the critical threshold of environmental change in a species with arbitrarily differentiated larval and adult stages (*η*_crit_(complex)) and contrasted that with the critical rate in an idealized species with no differentiation between its stages (*η*_crit_(simple), where *ρ* = 1, *B*_L_ = *B*_A_, *b*_L_ = *b*_A_, *γ*_L_ = *γ*_A_, *G*_L_ = *G*_A_). The ratio of the former to the latter – *η*_crit_(complex)/ *η*_crit_(simple) – quantifies the relative reduction in tolerance to environmental change that is caused by life‐history complexity. We note that this ratio does not preclude high (or low) likelihoods of persistence in both cases, but provides a clear point of contrast.

**Figure 1:**
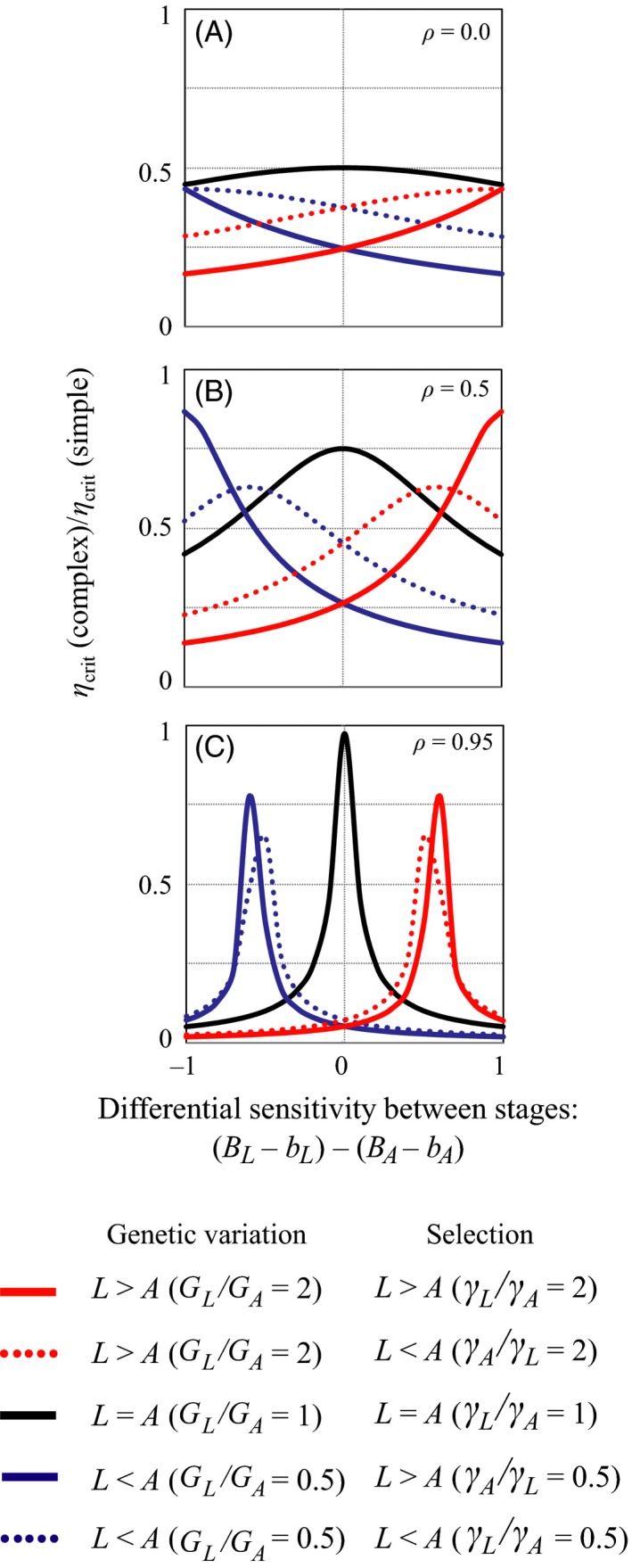
The relative sensitivities of species with simple versus complex life cycles to environmental change. Panel (A) shows the relative critical rate of environmental change of a species with no genetic correlation in tolerance to change between stages, panel (B) is for species with an intermediate genetic correction, and panel (C) is for a very high genetic correlation between life histories stages. Results use parameters (*G*_L_ + *G*_A_)/2 = 0.5; (*γ*_L_ + *γ*_A_)/2 = 0.04; ((*B*_L_ − *b*_L_) + (*B*_A_ − *b*_A_))/2 = 0.5.

Representative results, plotted in Fig. 1, illustrate three major factors that interact to increase the relative extinction susceptibility of species with complex life‐history stages. First, differences in net environmental sensitivity between stages (represented by (*B*_L_ − *b*_L_) − (*B*_A_ − *b*_A_) on the *x*‐axis of Fig. 1) can greatly inflate extinction susceptibility, particularly when genetic correlations between stages are strong and positive (*ρ* ≫ 0). Second, dimorphism in *G* between stages inflates extinction risk, particularly when there is less additive genetic variance in the stage that is more sensitive to environmental change (e.g. extinction risk is high when (*B*_L_ − *b*_L_)/(*B*_A_ − *b*_A_) > 1 and *G*_L_/*G*_A_ < 1). Third, low genetic correlations (small *ρ*) uniformly decrease the rate of environmental change that a species can tolerate. For example, when the genetic correlation is zero, the critical rate of environmental change in a species with complex life history, *η*_crit_(complex), decreases by at least a factor of two relative to the critical rate in a species with a simple life history. In each of these scenarios, differences in the strength of stabilizing selection between stages do not typically have a large effect on the results, provided the strength of stabilizing selection is modest to weak (*γ*_L_, *γ*_A_ ≪ 1).

### Genetic correlations and tolerance to environmental change

Genetic correlations between stages strongly influence the maximum rate of environmental change that a population can tolerate. The optimal genetic correlation between stages (hereafter *ρ*_max_, i.e. the value of *ρ* that maximizes the critical rate of environmental change (*η*_crit_)) depends on the relative amount of genetic variation in each stage (*G*_L_ versus *G*_A_), their relative strengths of stabilizing selection (*γ*_L_ versus *γ*_A_) and differences in the sensitivity of larvae and adults to environmental change ((*B*_L_ − *b*_L_) versus (*B*_A_ − *b*_A_)).

**Figure 2:**
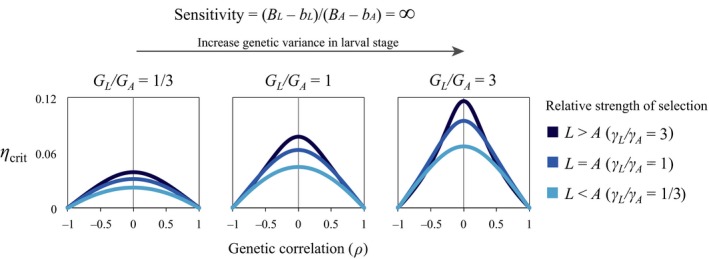
The predicted maximum rate of environmental change under which populations can persist (*η*_crit_) when the larval phase is sensitive to environmental change, but the adult phase is completely insensitive to the change (i.e. [*B*_L_ − *b*_L_]/[*B*_A_ − *b*_A_] = ∞). Note that negative genetic correlations between the adult and larval phase always decrease the maximum rate of environmental change that still allows population persistence. Increasing genetic variance in the larval phase and increasing the strength of stabilizing selection on the larval phase increases the population's capacity to tolerate environmental change when genetic correlations are relatively low. Results use parameters (*G*_L_ + *G*_A_)/2 = 0.5; (*γ*_L_ + *γ*_A_)/2 = 0.04; ((*B*_L_ − *b*_L_) + (*B*_A_ − *b*_A_))/2 = 0.5.

The optimal genetic correlation, *ρ*_max_, is only zero when one of the life‐history stages is completely insensitive to environmental change. In this case, one stage is experiencing a shift in the optimal trait value while the other experiences no change whatsoever. A nonzero genetic correlation in this instance generates maladaptation in the stage with a stable optimum (i.e. by displacing it from its optimum), while simultaneously slowing the adaptive evolutionary response in the stage with a moving optimum. Thus, the rate of adaptation is maximized when the two life‐history stage are genetically uncoupled (Fig. 2).

**Figure 3:**
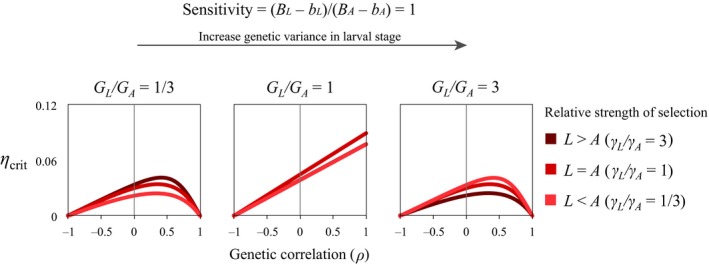
The predicted maximum rate of environmental change under which populations can persist (*η*_crit_) when the larval phase and the adult phase are equally sensitive to environmental change (i.e. [*B*_L_ − *b*_L_]/[*B*_A_ − *b*_A_] = 1). Note that when the level of genetic variation in the adult and larval phases is equal, *η*_crit_ increases with genetic correlations. When the level of genetic variation in each phase is unequal, *η*_crit_ is maximized at a positive but intermediate genetic correlation. Results use parameters (*G*_L_ + *G*_A_)/2 = 0.5; (*γ*_L_ + *γ*_A_)/2 = 0.04; ((*B*_L_ − *b*_L_) + (*B*_A_ − *b*_A_))/2 = 0.5.

**Figure 4:**
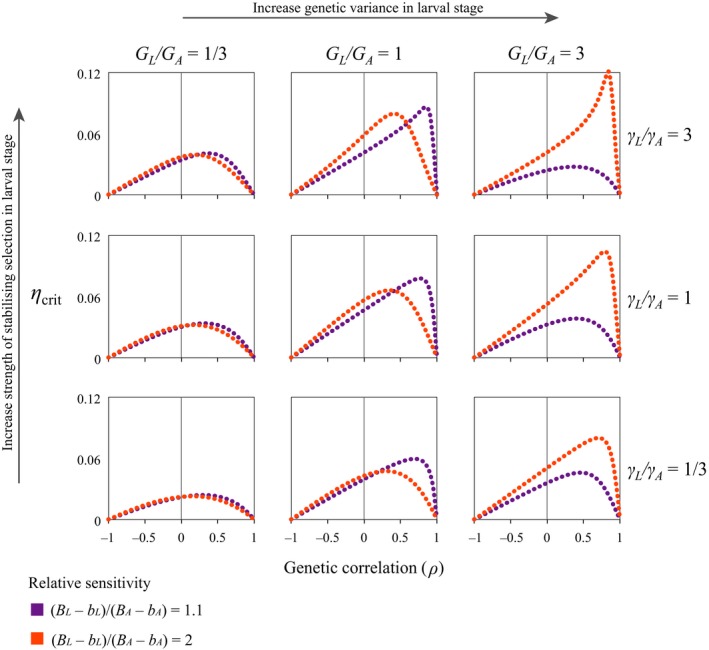
The predicted maximum rate of environmental change under which populations can persist (*η*_crit_) when the larval phase is more sensitive to environmental change than the adult phase, but the adult phase is still somewhat sensitive (i.e. [*B*_L_ − *b*_L_]/[*B*_A_ − *b*_A_] = 1.1 or 2). Note that the highest (*η*_crit_) is achieved when there is more genetic variation in the larval phase, and genetic correlations between the adult and larval phase are very high but less than 1. Results use parameters (*G*_L_ + *G*_A_)/2 = 0.5; (*γ*_L_ + *γ*_A_)/2 = 0.04; ((*B*_L_ − *b*_L_) + (*B*_A_ − *b*_A_))/2 = 0.5.

A positive genetic correlation between adult and larval traits can increase the capacity of a population to cope with environmental change as long as larval and adult optima move in the same direction (i.e. (*B*_L_ − *b*_L_)(*B*_A_ − *b*_A_) > 0). When there are no stage differences in selection or genetic variance, then *ρ*_max_ becomes unity (Fig. 3, middle panel; i.e. when *B*_L_ = *B*_A_, *b*_L_ = *b*_A_, *γ*_L_ = *γ*_A_ and *G*_L_ = *G*_A_, then *η*_crit_ is maximized at *ρ* = 1). In the presence of stage differences in selection, plasticity or genetic variance, tolerance to environmental change is maximized at an intermediate genetic correlation (0 < *ρ*_max_ **< 1; see Figs 2, 3, 4).

In general, differential sensitivity of larvae and adults to environmental change reduces *ρ*_max_ (*ρ*_max_ decreases as (*B*_L_ − *b*_L_)/(*B*_A_ − *b*_A_) deviates further away from unity).

### Relative genetic variance in each life‐history stage

The capacity of populations to cope with change generally increases when there is relatively more genetic variance in the more sensitive stage (Figs 2, 3, 4). Assuming larval stages are more sensitive (|(*B*_L_ − *b*_L_)/(*B*_A_ − *b*_A_)| > 1), those populations that can persist with the highest rate of environmental change are those in which genetic variance in the larval stage is much greater than genetic variance in the adult stage.

Genetic correlations and relative magnitudes of genetic variance interact strongly, such that when genetic variances are equal and sensitivities are equal, perfect correlations of 1 are favoured, otherwise values slightly less than 1 are favoured (Fig. 3).

### Relative sensitivity of the larval and adult stages

The consequences of differences in the sensitivity of larval and adult stages to change depend on the relative magnitude of genetic variance in each stage and the genetic correlation between stages (Figs 3 and 4). When there is less genetic variation in the larval stage relative to the adult stage, regardless of the genetic correlation, increasing the sensitivity of the larval stage decreases the capacity of populations to cope with change.

When there is equivalent genetic variation in both the larval and adult stages, populations where larvae are similar to adults in their sensitivity to change than adults tend to have greater capacity to cope with change than those where larvae are more sensitive, but genetic correlations and differences in stabilizing selection between larval and adult stages alter this tendency (Fig. 3). When genetic correlations between stages are intermediate, populations in which larvae that are much more sensitive to change than adults have the greatest capacity to cope with change (Fig. 4). When genetic correlations approach 1, populations where adults and larvae are similarly sensitive have the greatest capacity to cope with change.

When genetic variation in the larval stage is much greater than the genetic variation in the adult stage (as seems likely in most instances, although this awaits empirical confirmation), populations in which larvae are also much more sensitive to change have the greatest capacity to cope with change, particularly when genetic correlations are higher (but less than perfect). When larvae are more sensitive to change and correlations are high, increasing the relative strength of stabilizing selection on the larval stage generally increases the capacity of populations to cope with change (Fig. 4).

## Discussion

### How do complex life cycles hamper adaptation to change?

Across the parameter space that we explored, our model predicted that increasing the complexity of the life history decreased the potential for species to cope with environmental change. This finding matches general theory on the evolutionary costs of complexity. As argued by Blows (2007) and Walsh and Blows (2009), increasing the number of distinct genetic dimensions in which a population varies tends to reduce evolutionary potential unless orientation of the entire genetic covariance G matrix is well aligned with the vector of selection. Otherwise, there is limited genetic variation in the dimension in which selection and adaptation are constrained. While we only explored two life‐history stages, some marine organisms have more than five distinct life‐history stages, all occupying different habitats (Marshall and Morgan 2011). We suspect that increasing the number of life‐history stages should decrease the evolutionary potential to cope with environmental change, unless genetic correlations among stages are fortuitously aligned relative to selection. Overall, our model would predict that organisms with complex life histories are more likely to be susceptible to environmental change than species with simple life histories and that such species are more at risk of extinction in the face of future global change.

### What parameters should empiricists prioritize measuring?

Our model predicts that the capacity for marine organisms with complex life cycles to cope with climate change depends on a few key biological parameters, some of which are better understood than others. The relative sensitivity of fitness in the larval and adult stages to environmental stress is key, but we know remarkably little about this parameter because, as pointed out by others (Przeslawski et al. 2015), few studies incorporate global change stressors on both larval and adult phases. Instead, most studies examine either larvae or adults. Nevertheless, most meta‐analyses indicate that larval stages are much more sensitive than adult stages (Przeslawski et al. 2015), and from this perspective, a twofold difference in susceptibility to stress between stages used in our model seems conservative. Given the relative sensitivity of the model to this parameter, and the possibility of experimenting on larval and adult stages in many taxa, we suggest that future studies consider the effects of global change stressors on components of fitness (such as survival and reproduction) across both the adult and larval phase within the same study such that we can use more accurate parameters going forward. We should note that our parameter of relative sensitivity to stress serves as a good proxy for how different rates of environmental change (e.g. in the case of species where larvae inhabit fast changing surface waters and adults inhabit relatively unchanging deeper seas) will affect evolution. In this instance, greater sensitivity in the larval stage serves as a proxy for larvae experiencing faster rates of environmental change (importantly though, this is only a proxy, we did not explicitly model rates of change, only how optima change due the same change). Our model predicts that the genetic correlations between stages and the relative genetic variation within each stage will be crucial to determining the evolutionary capacity of such species to cope with future change.

The relative magnitude of genetic variation in traits between stages strongly affected the capacity of species to cope with change. When the more sensitive larval stage has greater genetic variation, higher rates of environmental change can be withstood compared to when the adult stage has more genetic variation. Estimates of additive genetic variation in stress resistance traits are exceedingly rare. Indeed, we know of only a few estimates of genetic variation across the life‐history more generally. In those few studies, there are no clear patterns: some studies show no differences in genetic variation across stages (Levin et al. 1991; Watkins 2001), while others show much more variation during the larval stage relative to the adult stage (Aguirre et al. 2014). Genetic variation in standardized traits across the life history is relatively straightforward to estimate in some species (Lynch and Walsh 1998; Munday et al. 2013), and we recommend that future studies pursue this path.

Genetic correlations in stress resistance among stages also profoundly influenced our predictions regarding the capacity for marine organisms to cope with environmental change. A positive genetic correlation between life‐history stages tends to increase the capacity of populations to cope with change relative to no (or negative) correlations among stages (a finding that is in keeping with multivariate, geometric view of adaptation; Blows 2007; Walsh and Blows 2009). Assuming that adults are at all sensitive to environmental change, because both trait optima change in the same direction in response to global change (albeit possibly at different rates), the genetic correlations between stages create complementary responses to selection, accelerating evolution and increasing the potential for evolutionary rescue. This finding suggests that while the sensitivity of larvae to environmental change may create a demographic bottleneck in the shorter term (Byrne 2011, 2012), if species survive such bottlenecks, positive genetic correlations among stages could facilitate more rapid evolution in the longer term. Estimates of genetic covariation across life‐history stages are rare generally (Marshall and Morgan 2011) and, as far as we are aware, nonexistent for responses to stress. Nevertheless, there is a reasonably long history of estimating genetic correlations across life‐history stages in some marine organisms (Levin et al. 1991; Evans et al. 2007), and apart from statistical and logistical issues, their estimation is possible though challenging. Aguirre et al. (2014) recently provided a new statistical framework for formally estimating the extent of genetic correlations among life‐history stages for marine invertebrates, and we recommend such an approach in future studies.

### What characteristics make species most or least vulnerable to environmental change?

Managers need to know what species are likely to be the most vulnerable to global change and which are likely to be relatively robust in order to prioritize management efforts and identify problems early (Bottrill et al. 2008). Some general rules of thumb already exist. For example, minimizing selection bottlenecks, maximizing genetic variation in the standing population and enhancing effective population size will all maximize the rate at which populations will evolve and therefore cope with climate change (Hoffmann and Sgro 2011; Pandolfi et al. 2011; Sgro et al. 2011). Our model predicts that certain combinations of characteristics will render species more or less vulnerable to environmental change. Species with relatively less genetic variance in the sensitive larval stage are predicted to be the least resilient to change, and for these species, strong genetic correlations (both positive and negative) will actually reduce resilience even further. Negative correlations between traits in the larval and adult stage also reduce the evolutionary capacity to cope with climate change across much of the parameter space that we explored. These results have intuitive appeal. When there is little genetic variation in the sensitive larval stage, there is little scope for adaptation (Walsh and Blows 2009). If adults are relatively insensitive to environmental change, any genetic correlation between adult and larvae slows adaptation further. In this regard, any genetic correlation between adults and larvae when adults are insensitive to change acts as conduit by which adaptation is slowed. We also found that negative genetic correlations between adults and larvae tended to reduce adaptive capacity. While we are unaware of any formal estimates of genetic correlation in stress resistance among marine life‐history stages specifically, more general explorations show that negative genetic correlations across the life history do occur in marine invertebrates (Levin et al. 1991; Evans et al. 2007; Aguirre et al. 2014). If such negative correlations extend to resistance to global change stressors, then the capacity for such species to cope with change will be extremely limited.

Some combinations of species traits maximize the evolutionary capacity of species to cope with climate change. Our model predicts that those species with relatively more genetic variance in the larval stage and relatively large differences in the sensitivity of larvae and adults will have the greatest evolutionary capacity to cope with environmental change, particularly when genetic correlations are very high (although not perfect). Some of these results make sense – when there is ample genetic variation in the most sensitive stage, then adaptation should proceed unimpeded. A less straightforward explanation is apparent for why larger differences in sensitivity between adults and larvae can still yield high evolutionary resilience, particularly when genetic correlations are moderately high (although less than 1). The explanation lies in the fact that when genetic variance in the adult phase is low, then the only way in which adaptation can proceed rapidly is when all of the selection are concentrated on the larval phase and a genetic correlation between the adult phase and the larval phase ‘drags’ the trait distribution of the adult phase along.

### Application to nonmarine organisms with complex life cycles

Overall, we found that complex life histories where larvae and adults exhibit differential sensitivity to environmental change hamper evolutionary responses to change. Our results are particularly relevant to marine organisms because it is this group where early life‐history stages are particularly sensitive relative adults. Nevertheless, our results have relevance to other systems where different life‐history stages have differential sensitivity to environmental change, either because one stage is more sensitive (e.g. insects and freshwater fish; Lee and Denlinger 1985; Williams et al. 1986; Munkittrick and Dixon 1989; Mitchell et al. 2013), or because one stage experiences more environmental change than the other. For example, parasites that alternate between invertebrate hosts that are temperature‐conforming and vertebrate hosts that are temperature regulating may suffer similar constraints as to those modelled here. An important next step will be to estimate the key genetic and phenotypic parameters modelled in our study for a wider range of organisms.
